# Dynamic quantitative monitoring of cerebrospinal fluid monoamine neurotransmitter markers during the modeling process of chronic stress‐induced depression in monkeys (*Macaca mulatta*)

**DOI:** 10.1002/brb3.3636

**Published:** 2024-08-21

**Authors:** Siyu Li, Xiaoli Feng

**Affiliations:** ^1^ Department of Physiology, Faculty of Basic Medical Science Kunming Medical University Kunming Yunnan China; ^2^ Key Laboratory of Animal Models and Human Disease Mechanisms of the Chinese Academy of Sciences & Yunnan Province, Kunming Institute of Zoology Chinese Academy of Sciences Kunming Yunnan China; ^3^ Institute of Neuroscience Kunming Medical University Kunming Yunnan China

**Keywords:** chronic stress, depression, early adversity, monoamine transmitters

## Abstract

**Background:**

Depression is known as the “mental cold” and is also considered a major cause of disability worldwide. It is estimated that over 300 million people worldwide suffer from severe depression, equivalent to 4.4% of the world's population. The monoamine hypothesis of depression predicts the underlying pathophysiological mechanisms of depression, but in‐depth research has failed to find convincing evidence.

**Method:**

In this study, we will dynamically and strictly quantitatively monitor the concentration changes of monoamine transmitters in the cerebrospinal fluid (CSF) of macaques, based on our previous work. In the experiment, timed and quantitative collection of CSF samples from macaques was performed and the concentration of monoamine transmitters was determined.

**Result:**

The results showed that after 2 months of chronic stress, the concentrations of high vanillin acid (HVA) and 3,4‐dihydroxy‐phenylacetic acid were significantly higher in the maternal separation (MS) group, whereas there was no significant difference in dopamine and 5‐hydroxyindoleacetic acid.

**Conclusion:**

This study is the first to observe the long‐term dynamic relationship between early adversity, chronic stress, adolescent depression, and CSF monoamine concentrations. The research suggests that MS and chronic stress play an undeniable role in the pathogenesis of depression and that concentrations of HVA and dihydroxyphenylacetic acid are likely to serve as early markers of depressive‐like symptoms in macaques.

## Graphical abstract

We observe the long‐term dynamic relationship between early adversity, chronic stress, adolescent depression, and CSF monoamine concentrations. The research suggests that MS and chronic stress play an undeniable role in the pathogenesis of depression, and HVA and DOPAC are likely to serve as early markers of depressive‐like symptoms in macaques.

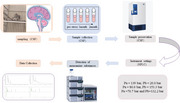

## 1 INTRODUCTION

Depression is known as the “mental cold” and is considered the main cause of disability worldwide, even leading to suicide (Lopez & Murray, 1998; Marwaha et al., 2023). It is estimated that over 300 million people worldwide suffer from severe depression, equivalent to 4.4% of the world's population (Chodavadia et al., 2023), and among the elderly, the incidence rate has reached an astonishing 35.1% (Cai et al., 2023). In 2008, the World Health Organization identified major depression as the third greatest contributor to the global burden of disease and projected that it would become the leading cause by 2030 (Malhi & Mann, 2018).

Numerous epidemiological and clinical research findings suggest that early adversity (EA) is a widely accepted risk factor for the onset of depression in later life (Goff & Tottenham, 2015). EA encompasses various forms of stress encountered in early life, including physical, sexual, and emotional abuse, parental neglect or loss, substance abuse, and incarceration by parents or caregivers. Such experiences can disrupt physiological and psychological functions and have a deleterious impact on physical well‐being (Brenhouse et al., 2019; Brown et al., 2009). Research indicates that children who experience abuse and other negative childhood events, known as adverse childhood experiences, are at a heightened risk of developing a range of diseases, such as cancer, liver disease, substance abuse, and depression (Hunt et al., 2017). Another study also found a strong dose‐dependent relationship between poor childhood experience scores and the probability of lifelong and recent depressive disorders (Chapman et al., 2004). However, not all individuals who have experienced EA ultimately exhibit depression or depressive like behavior. In fact, in our previous research on nonhuman primates, rhesus monkeys that have experienced EA (usually simulated using maternal separation [MS] in animal models), although considered an ideal animal model for simulating EA, did not exhibit the internationally recognized typical depressive phenotype (Hudding up) (Feng et al., 2011). It is apparent that MS results in prolonged neurological alterations; however, it does not directly instigate a depressive phenotype. In essence, macaques experiencing MS still require an external trigger factor to develop depressive symptoms.

Regarding the external trigger factors of depression, it is internationally recognized that chronic stress is an undeniable external trigger factor. Studies have shown that long‐term exposure to chronic stress increases susceptibility to anxiety, depression, and other emotional disorders (Cohen et al., 2007; McEwen & Morrison, 2013; Nemeroff, 2004). Therefore, based on the team's aforementioned MS model in macaques, this study introduces an external factor of chronic stress to prove that MS after birth can increase the risk of depression in macaques and is a reliable etiological animal model of depression. This part of the work has also been internationally recognized (Zhang et al., 2016).

A dependable animal model for pathogenic depression is essential in uncovering the disease's associated mechanisms. The monoamine hypothesis is among the widely accepted theories in explaining the mechanism behind depression (Hirschfeld, 2000; Jiang et al., 2022; López‐Muñoz & Alamo, 2009). The close connection between the monoamine system and depression has been confirmed from multiple perspectives. The monoamine pathway has always been the center of psychopharmacology and has been targeted by multiple drugs from multiple perspectives (López‐Muñoz & Alamo, 2009). A research study investigating the functional connectivity of the default mode network (DMN) in depressive states has demonstrated that 5‐hydroxytryptamine norepinephrine reuptake inhibitors resulted in improved DMN hyper‐connectivity in rats (van Wingen et al., 2014). Meanwhile, pertinent research has indicated that dopamine (DA) reuptake inhibitors may have antidepressant properties by boosting DA availability in striatal synapses (Argyelán et al., 2005). There are also studies providing evidence of reduced levels of high vanillin acid (HVA) (Ogawa & Kunugi, 2019) and 5‐hydroxyindoleacetic acid (5‐HIAA) (Fu et al., 2023) in the cerebrospinal fluid (CSF) of patients with depression, which are metabolites of catechol amines.

Numerous studies have indicated a strong correlation between depression and a reduction in levels of monoamine neurotransmitters (serotonin, norepinephrine, 3,4‐dihydroxy‐phenylacetic acid [DOPAC], and/or DA) in the central nervous system. Nevertheless, research findings are inconsistent regarding changes in monoamine transmitters in depression, with some even contradicting one another. Current research reports indicate that in patients with depression, there are reports of decreased and increased (Ogawa & Kunugi, 2019; Post et al., 1986) and increased (Aberg‐Wistedt et al., 1985; Jori et al., 1975) concentrations of 5HIAA and HVA in the CSF. The inconsistent research findings on changes in CSF monoamines in depression may be due to: (1) There may be differences in the regulatory ability and manner of the DA system among rodents, nonhuman primates, and humans due to differences in experimental subjects (Meador‐Woodruff et al., 1994). Therefore, in this study, nonhuman primates with the closest brain development to humans were used as experimental subjects; (2) The collection sites of monoamines are different. Some studies measure monoamine levels in blood or urine, whereas in depression, as a brain dysfunction disease, it is more intuitive to measure monoamine levels in CSF. Therefore, this study plans to use CSF as the sample; (3) Due to different collection times, there may be differences in the levels of monoamine neurotransmitters at different times, such as early, middle, and late stages of depression. To date, most studies have used post‐onset sampling. It should be noted that continuous and dynamic monitoring of monoamine levels during and after the onset of depression can more comprehensively measure the role of changes in brain monoamine levels in the onset of depression. This study used a high‐performance liquid chromatograph (HPLC) to continuously and dynamically monitor the levels of monoamine transmitters in the CSF of macaques.

In this study, we used the rhesus monkey, a nonhuman primate that is closest to humans in terms of brain development. Based on previous research, the concentration of monoamine transmitters in the CSF was measured dynamically and strictly quantitatively from the beginning to the end of the chronic stress application. The relationship between MS, chronic pressure, and changes in the content of monoamine transmitters in the CSF was investigated, and its profound correlation was further explored. Our research can provide some theoretical basis for the possible biochemical mechanisms of depression.

## 2 MATERIALS AND METHODS

### 2.1 Subjects

Eight newborn male rhesus monkeys (*Macaca mulatta*) were selected and randomly divided into two groups: MS (*n* = 4) and mother reared (MR, *n* = 4). The MS newborns were separated from their mothers at birth and reared solely for the first month in an incubator. Then, the MS infants were paired off and lived together in a steel cage (0.74 × 0.71 × 0.74 m^3^) for about 6 months. The MR monkeys had been living with their mothers for 7 months since they were born. After that, both the MS and MR monkeys were moved into a connected indoor (2.61 × reared 2.46 × 2.58 m^3^) and outdoor (2.67 × 2.66 × 2.67 m^3^) colony and lived together.

All animal procedures have been approved by the National Institute of Health's Guidelines for the Care and Use of Experimental Animals, and all 8 macaques (*n* = 8) used in this experiment have been authorized by the Animal Care and Use Committee of Kunming Animal Research Institute. It has passed the ethical review of animal experiment welfare of Kunming Institute of Zoology, Chinese Academy of Sciences.

### 2.2 Experimental design

These eight monkeys were reared together for a period of time prior to chronic stress and then placed in separate cages. After a 1‐month adaptation period, several unpredictable chronic stressors (spatial restriction, intimidation, prolonged illumination, and fasting) were randomly administered. Before the administration of the chronic unpredictable stressors, baseline levels of monoamines transmitters were measured in the CSF of the rhesus monkeys until the end of the chronic unpredictable stressors. The concentration of monoamines in the CSF was quantified dynamically and rigorously throughout the process. With the exception of long‐term illumination, the illumination is maintained at a 14/10 light/dark cycle and the temperature at 22°C. Unless the day of fasting, the monkeys were provided sufficient commercial monkey biscuits twice a day and fruits or vegetables once daily with tap water ad libitum.

### 2.3 Sample collection

**FIGURE 1:**
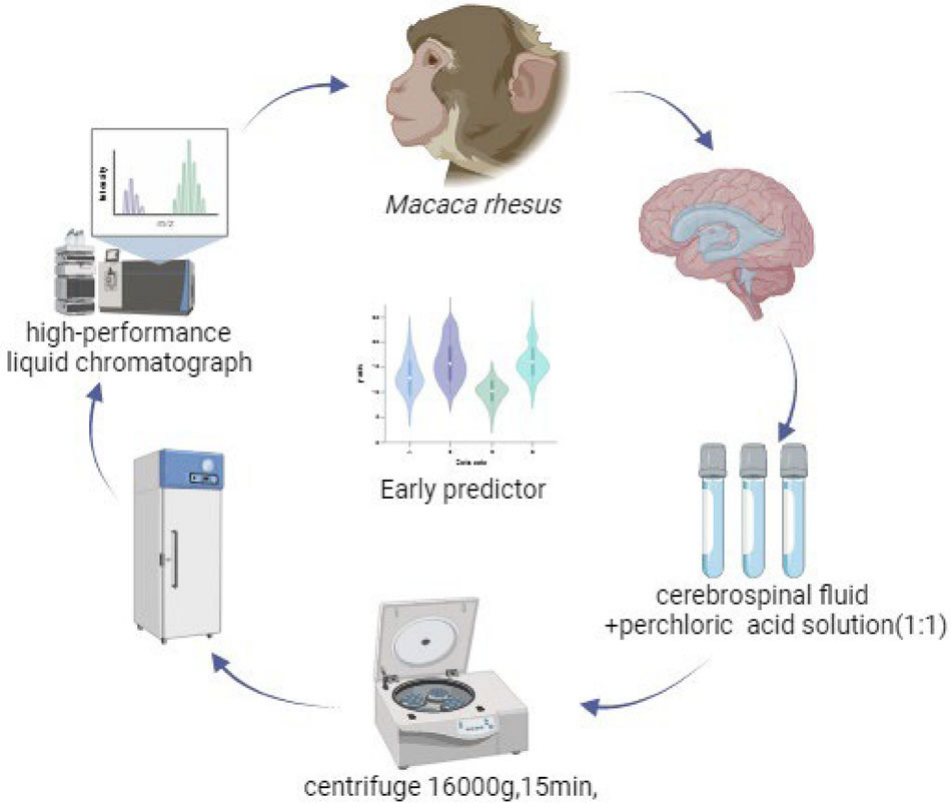
This figure shows the process of collecting cerebrospinal fluid (CSF) samples. Collect CSF samples from eight macaques (*Macaca mulatta*), mix them evenly with 0.1 mol/L perchloric acid solution in a 1:1 ratio, centrifuge at a speed of 16,000 *g* for 15 min in a high‐speed centrifuge, store them in a −80°C refrigerator, and finally perform electrochemical detection of the concentration changes of high vanillin acid (HVA), 5‐hydroxyindoleacetic acid (5‐HIAA), dopamine (DA), and 3,4‐dihydroxy‐phenylacetic acid (DOPAC) in the CSF using high‐performance liquid chromatograph (HPLC).

First, anesthetize the animals with a dose of 0.08 mL/kg of Zoletil 50. Before lumbar puncture, young monkeys must be deprived of food and water for 2 h to prevent gastric reflux caused by anesthesia. The puncture site is usually located in the upper or lower intervertebral space of the fourth lumbar vertebral process in monkeys (i.e., the 3rd to 4th or 4th to 5th lumbar vertebral spaces) and the 7th intervertebral space in humans. Store the CSF in 2–3 sterile cryopreservation tubes, approximately 300 µL each. A 1.5 mL EP tube was then filled with a 1:1 ratio of 0.1 mol/L perchloric acid solution, placed in a centrifuge at 4°C, centrifuged at 16,000 *g* for 15 min, packed, and stored in a −80°C refrigerator. It should be noted that the collected CSF must be clear, without blood color, turbidity, or pale yellow. The collected CSF should be rapidly frozen and stored in liquid nitrogen (Figure 1).

### 2.4 HPLC

**FIGURE 2:**
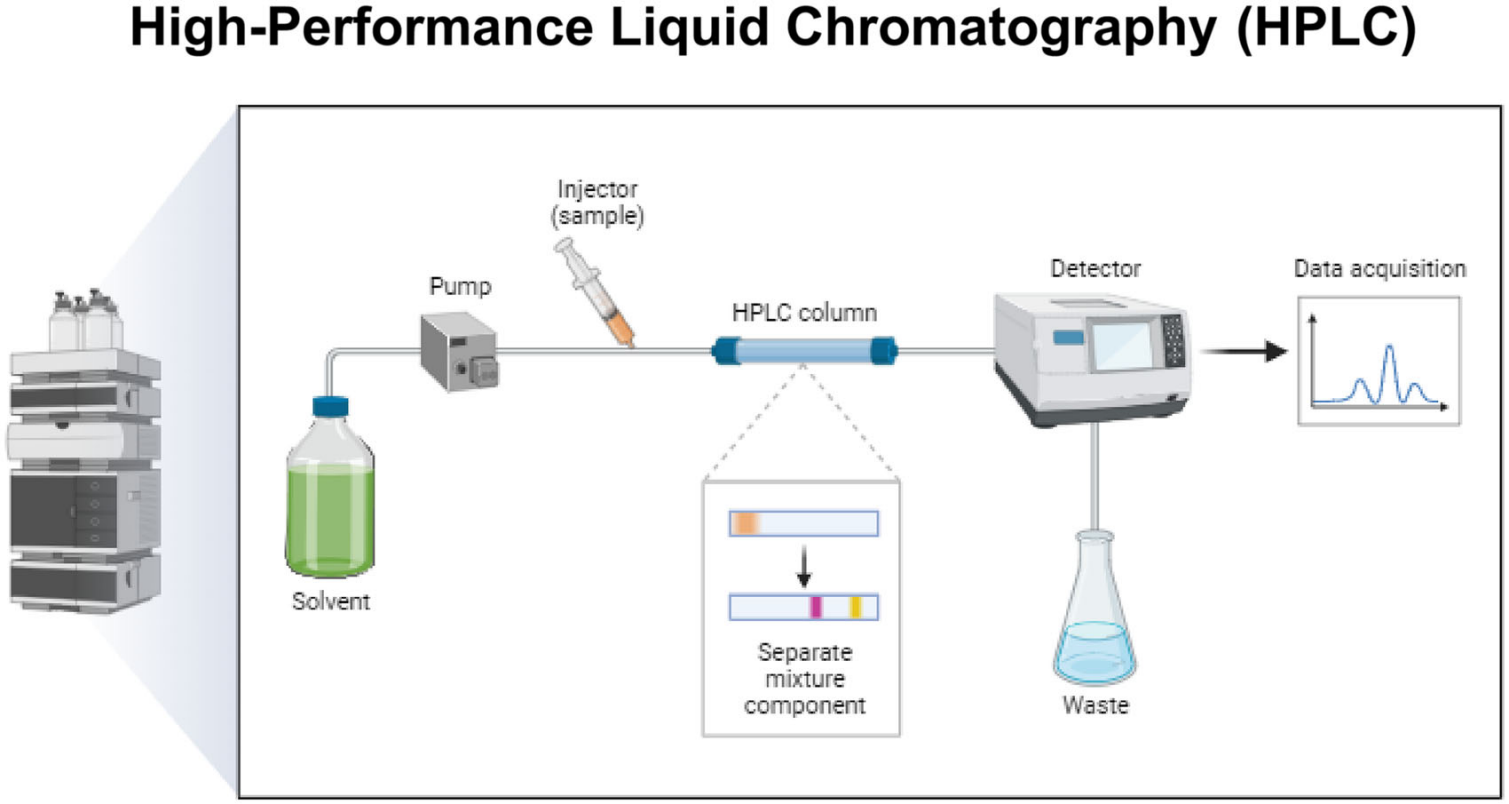
Flowchart for the determination of monoamines transmitters in cerebrospinal fluid (CSF) by high‐performance liquid chromatograph (HPLC). The concentration of four substances, high vanillin acid (HVA), 5‐hydroxyindoleacetic acid (5‐HIAA), dopamine (DA), and 3,4‐dihydroxy‐phenylacetic acid (DOPAC), in CSF was detected by HPLC electrochemical detection. Overall, 20% and 80% methanol water were used for detoxification, needle washing, and pump head cleaning. Subsequently, mobile phases of long and short columns were prepared separately, and parameters were set for monitoring and analysis.

HVA, 5‐HIAA, DA, and DOPAC were detected electrochemically by HPLC (Scheinin et al., 1983). First prepare 500 mL of 20% methanol‐water and 200 mL of 80% methanol‐water and detoxify with ultrasound, then replace the 20% methanol‐water for washing the needles and cleaning the pump heads, replace the 80% methanol‐water for running the column, and switch on the power in turn. The start‐up flow rate of the pump is set to 40 µL/min, and 80% methanol‐water is used to purge the bubbles in front of the column (PA = 13.9 bar and PB = 20.0 bar). When the bubbles have been completely removed, the two columns are connected to the system. Then replace the column with 20% methanol‐water, pump to pressure (PA = 80.0 bar and PB = 153.3 bar), prepare the mobile phase (ultrasonic degassing) of the long and short column respectively, replace the mobile phase, pump the system to mobile phase pressure (PA = 70.7 bar and PB = 132.2 bar) overnight, prepare the injection, and draw the standard curve once the baseline is stable. Finally, add the sample to be tested to the sample tray. When the chromatogram is displayed, calculate the content of the substance to be tested in the sample and generate a report (Figure 2).

### 2.5 Statistical data analysis

**FIGURE 3:**
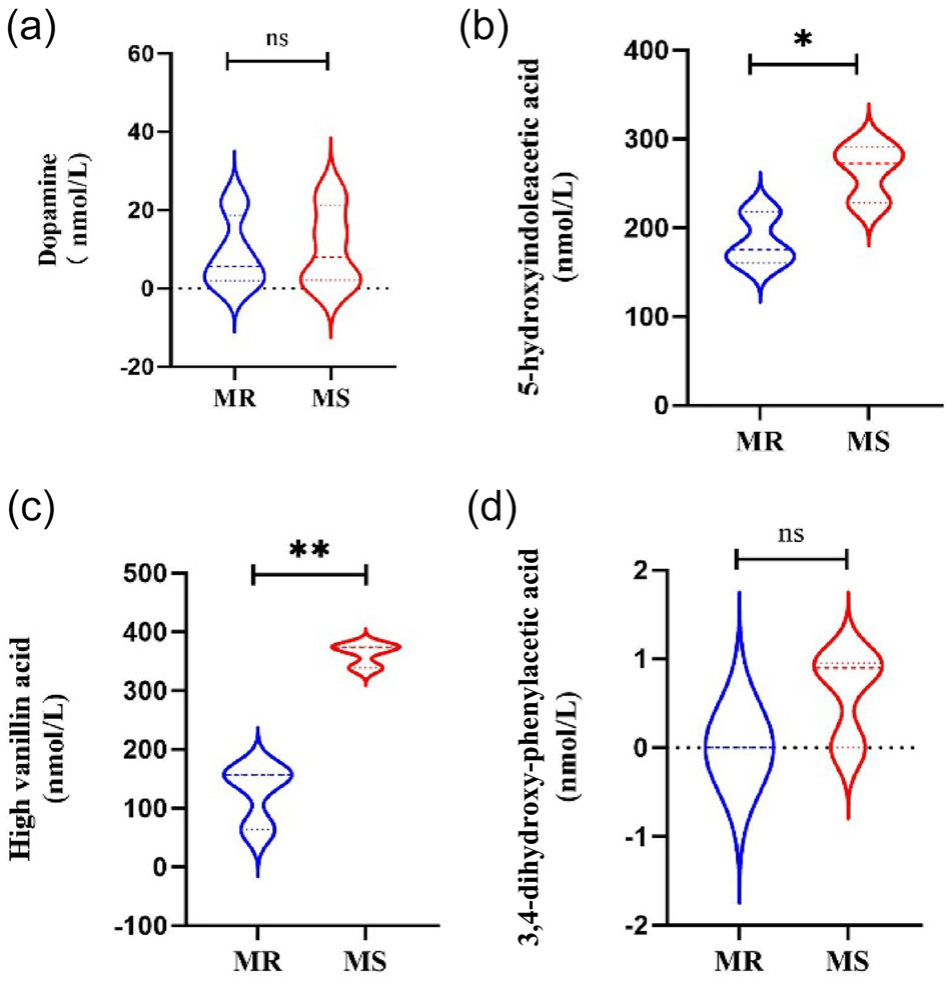
The concentration changes of dopamine (DA), 5‐hydroxyindoleacetic acid (5‐HIAA), high vanillin acid (HVA), and 3,4‐dihydroxy‐phenylacetic acid (DOPAC) in the cerebrospinal fluid (CSF) of macaques after experiencing maternal separation (MS) (before introducing chronic stress). (a) After experiencing MS, it was found that there was no significant change in the concentration of DA in the CSF of macaques in the MS group compared to the MR group (unpaired *t* test, *t* = .2415, *p* = .8172). (b) After experiencing MS, it was found that the concentration of 5‐HIAA in the CSF of macaques in the MS group was significantly higher than that in the MR group (unpaired *t* test, *t* = 3.116, ^*^ *p* = .0357). (c) After experiencing MS, it was found that the concentration of HVA in the CSF of macaques in the MS group was significantly higher than that in the MR group (unpaired *t* test, *t* = 7.118, ^**^ *p* = .0021). (d) After experiencing MS, it was found that there was no significant change in the concentration of DOPAC in the CSF of macaques in the MS group compared to the MR group (unpaired *t* test, *t* = 1.997, *p* = .1165).

**FIGURE 4:**
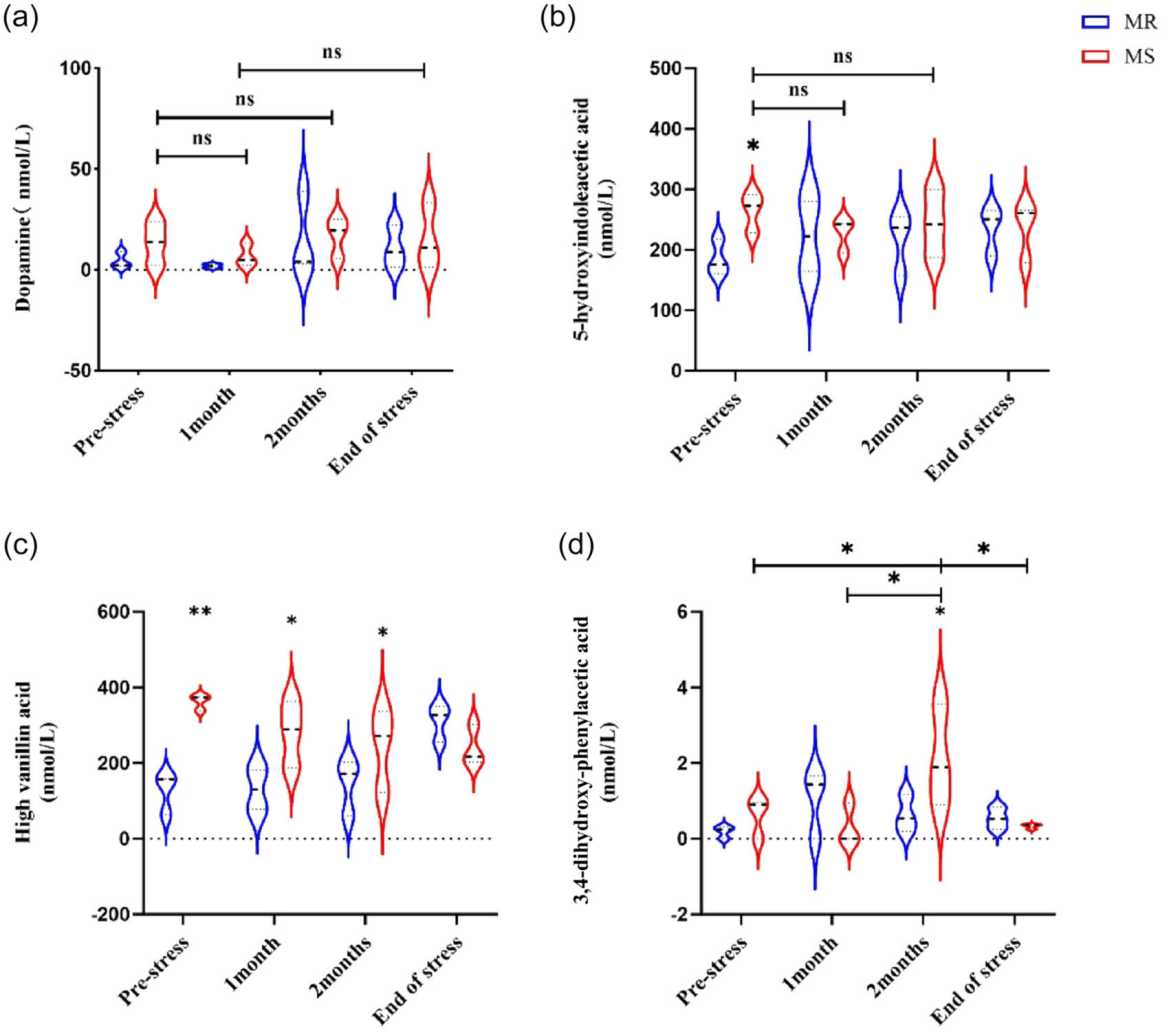
After 2 months of chronic stress, continuously monitor the concentration changes of dopamine (DA), 5‐hydroxyindoleacetic acid (5‐HIAA), high vanillin acid (HVA), and 3,4‐dihydroxy‐phenylacetic acid (DOPAC). (a) After 2 months of chronic stress, there was no significant change in DA concentration in the maternal separation (MS) group of macaques compared to before experiencing chronic stress (Tukey's multiple comparisons test, LSR = −3.455, *p* > .9999). (b) After 2 months of chronic stress, there was no significant change in the concentration of 5‐HIAA in the MS group of macaques compared to before experiencing chronic stress (Tukey's multiple comparisons test, LSR = 21.19, *p* = .9974). (c) After experiencing 1 month of chronic stress, the concentration of HVA in the MS group macaques significantly increased compared to the MR group macaques (Tukey's multiple comparisons test, LSR = −141.6, ^*^ *p* = .0166), after 2 months of chronic stress, the concentration of HVA in the MS group macaques significantly increased compared to the MR group macaques (Tukey's multiple comparisons test, LSR = −98.41, ^*^ *p* = .0429). (d) Introducing 2 months of chronic stress, it was found that the concentration of DOPAC in the MS group showed a significant upward trend. After the end of 2 months of chronic stress (Tukey's multiple comparisons test, LS = −1.482, ^*^ *p* = .0469; LSR = −1.931, and ^*^ *p* = .0136), the concentration of DOPAC decreased (Tukey's multiple comparisons test, LSR = 1.789, ^*^ *p* = .0198).

Figure 3 uses unpaired *t*‐tests for statistical analysis of changes in monoamine concentration in CSF after MS, whereas Figure 4 uses Tukey's multiple comparison test to analyze the effect of chronic stress on monoamine concentration in CSF of rhesus monkeys. Before conducting statistical analysis, normality testing should be conducted. ^*^ *p* < .05, ^**^ *p* < .01, and ^***^ *p* < .001. All *p* values were generated by two‐tails tests. The difference was not statistically significant (*p* > .05).

## 3 RESULTS

### 3.1 Changes of DA concentration

Prior to the addition of chronic stress, there was no statistically significant difference in changes in CSF DA concentrations between the MS and MR macaques (Figure 3a, unpaired *t* test*, t* = .2415, *p* = .8172). When both groups of macaques were subjected to the same 2‐month chronic stress at a later stage, there was still no significant difference in DA concentration between the MS group and the MR group (Figure 4a, Tukey's multiple comparisons test, LSR = −3.455, *p* > .9999), and the level of CSF DA remained relatively stable.

### 3.2 Changes of 5‐HIAA concentration

Compared with the MR group, the concentration of 5‐HIAA in the CSF of macaques (MS group) that had experienced MS was significantly increased (Figure 3b, unpaired *t* test, *t* = 3.116, ^*^ *p* = .0357). After introducing the same 2‐month stress to both groups of macaques at a later stage, there was no significant change in the concentration of 5‐HIAA in the CSF of the MS group macaques compared to the MR group, and no significant change was observed compared to the previous concentration level of the MS group macaques (Figure 4b).

### 3.3 Changes of HVA concentration

Compared to the MR group, monkeys that had experienced MS had significantly elevated HVA levels prior to the addition of chronic stress (Figure 3c, unpaired *t* test, *t* = 7.118, ^**^ *p* = .0021). After introducing the same 2 months of stress to both groups of macaques, it was observed that HVA levels in the MS group were still significantly higher than those in the MR group (Figure 4c, Tukey's multiple comparisons test, LSR = −141.6, ^*^ *p* = .0166; LSR = −98.41, ^*^ *p* = .0429), whereas the changes in HVA concentration levels in the MR group were relatively stable.

### 3.4 Changes of DOPAC concentration

After experiencing MS, there was no significant change in the concentration of DOPAC in the CSF of the MS group compared to the MR group (Figure 3d, unpaired *t* test, *t* = 1.997, *p* = .1165); however, 2 months after the addition of chronic stress, the concentration of DOPAC in the CSF of the MS group macaques significantly increased compared to before, and the concentration also significantly increased compared to the MR group. After the end of chronic stress, the concentration of DOPAC in the MS group of macaques decreased significantly (Figure 4d).

## 4 DISCUSSION

CSF comes into contact with the brain's interstitial fluid and is largely isolated from the peripheral circulation by the blood–brain barrier (Strittmatter, 2013). Therefore, CSF is considered an ideal material to study biomarkers of the central nervous system. In this study, we found that the concentrations of HVA and 5‐HIAA in macaques after MS were significantly higher than those in the MR group (both *p* < .05), whereas there was no significant difference in DA and DOPAC between the MS and MR groups (*p* > .05). After 2 months of chronic stress, significant increases in CSF HVA and DOPAC concentrations were observed in the MS group of macaques (*p* < .05). Compared to the MR group, no significant changes in DA and 5‐HIAA concentrations were observed between the two groups. The relevant key factors, including onset time, monoamine accumulation, controlled patterns, and duration of EA and chronic stress, provide more reliable data on the relationship between EA, chronic stress, and depression.

In addition, previous studies have mostly focused on the relationship between EA and CSF monoamine concentrations in patients diagnosed with depression, but few studies have conducted long‐term dynamic studies of adolescent depression. This study is the first to dynamically monitor changes in CSF monoamine concentrations in macaques that have undergone MS under various unpredictable chronic stresses.

After the MS, the concentrations of HVA and 5‐HIAA in the CSF of the macaques increased significantly, whereas the concentrations of DA and DOPAC did not show any significant changes, in agreement with previous research by Jori et al. 1975, Scheinin et al. 1983, and others. This also provides indirect evidence for our previous research findings that MS may increase susceptibility to depression in macaques. Recently, a study showed a significant negative correlation between depression scores and CSF HVA in patients with depression (Faustman et al., 1991), which is inconsistent with our findings. Following chronic stress, the concentration of HVA and DOPAC in the CSF of the macaques increased significantly. And our previous research confirmed that this group of macaques showed significant depressive behavior ‐Hudding up (Zhang et al., 2016). It can be seen that the levels of HVA and DOPAC are likely to serve as early predictors of depressive symptoms in macaques (Ogawa & Kunugi, 2019; Ogawa et al., 2018).

It is worth noting that prior to the application of chronic stress, the monoamine transmitter concentrations of both groups of macaques, except for DA, showed a significant upward trend in HVA and 5‐HIAA. After adding unpredictable chronic stress as a triggering factor for 2 months, we observed significant changes in HVA and DOPAC concentrations but not in DA and 5‐HIAA concentrations, which is inconsistent with the results reported in some related studies (Aberg‐Wistedt et al., 1985; Post et al., 1986).

To this end, we propose the following explanations. First, it is reported that EA can have a significant impact on brain development, even on a whole brain scale. There is also preclinical evidence that EA may lead to long‐term disturbances in the DA nervous system transmission that persist into adulthood (Oswald et al., 2014). This may cause macaques that have experienced MS to show alterations in the DA response to stress during later chronic stress (Brake et al., 2004; Chocyk et al., 2011; Jahng et al., 2010), and the macaque brain may still be in the DA system disorder caused by EA; therefore, no significant changes in the concentration of monoamine transmitters (except for HVA and DOPAC) were observed. Second, a striking example is the research report published in nature by Chaudhury et al. (2013), which suggests that stressors of EA and chronic stress may regulate the inhibitory excitation pathway through different pathways, with different emphasis on brain regions, which may lead to inconsistent results in changes in the levels of some biochemical metabolites (Venzala et al., 2013). Macaques show certain differences in behavioral phenotypes when experiencing MS and chronic stress, which may correspond to different stress circuits as mentioned above. Therefore, we did not observe significant changes in the levels of HVA and 5‐HIAA in the MS and MR groups of chronically stressed macaques. In addition, studies in experimental animals and humans have shown that measurement of major monoamine metabolites provides an indicator of maternal neurotransmitter conversion. In clinical trials, measurement of metabolite levels in lumbar CSF may provide the most direct assessment of central neurotransmitter function. However, these measurements reflect overall central nervous system activity and cannot reflect local changes in neurotransmitter release.

Previous studies have shown that after 1 month of chronic stress application, the MS group of macaques showed a significant decrease in autonomous movement, whereas stereotyped and curled up behaviors significantly increased. Even after 2 months of chronic stress, the MS group exhibited abnormal behavioral phenotypes. However, for the MR group, there was almost no change in exercise levels from pre stress to 2 months after stress, such as stereotypes and social behavior. In addition, compared with the MR group of macaques, there were significant changes in cortisol levels and body weight (Zhang et al., 2016). The differences in HVA and 5‐HIAA concentrations between MS and MR macaques before the addition of chronic unpredictable stress are related to MS, whereas the significant differences in HVA and DOPAC concentrations between MS and MR macaques after the addition of chronic unpredictable stress may be related to external triggers (chronic stress). This may be related to a key brain region—the amygdala, as the amygdala projects to the DA and serotonin‐enriched areas (Asim, Wang, Waris et al., 2024), whereas previous studies have shown that in stress‐induced depression, the amygdala is closely related to changes in its neurotransmission (Asim et al., 2023), and enhanced neuronal activity in the basal lateral amygdala of GABA can alleviate stress‐induced depressive behavior (Asim, Wang, Chen et al., 2024). This suggests that changes in monoamine neurotransmitters in the CSF of macaques can be closely related to the amygdala, a brain region, after experiencing chronic stress. Of course, this requires further exploration to explain the specific connections between them.

Specifically, the differences in CSF monoamine concentrations may result from different stress environments (MS and chronic stress). Similarly, differences in stress environments may lead to behavioral and cognitive differences (Zhang et al., 2016). This theory does not negate our main findings that EA and later chronic stress may be important factors that cannot be ignored in the onset of depression. In addition, our previous studies have confirmed that macaques experiencing chronic stress following MS show significant depressive behavior (Hudding up), and there are also significant differences in the levels of HVA and DOPAC in the CSF of these macaques. HVA and DOPAC are likely to serve as early markers of depressive behavior in macaques. In conclusion, we believe that our data can provide valuable insights into the underlying biochemical mechanisms of depression.

There are limitations to this study, as we did not collect data from magnetic resonance imaging throughout the entire study. Therefore, we are unable to clarify the brain region associations involved in EA, chronic stress, behavior, and changes in CSF monoamine concentration, which requires further exploration. In addition, after the establishment of the macaque depression model (i.e., after removing chronic stress), we only observed the following month without long‐term dynamic tracking, and long‐term monitoring may search for new therapeutic targets and directions for the treatment of depression.

## 5 CONCLUSION

We have confirmed that EA and chronic stress may be important factors that cannot be ignored in the development of depression, not only in adults but also in adolescents. EA and chronic stress have an impact on brain development, which is also reflected in changes in the concentration of monoamine transmitters in the CSF. However, more research is needed to clarify the relationship between the trend of changes in the concentration of monoamine transmitters in the CSF and EA, as well as the presence of chronic stress. However, based on previous research and the results of this study, we speculate that HVA and DOPAC concentrations are likely to serve as early markers of depressive symptoms in macaques.

## AUTHOR CONTRIBUTIONS

**Siyu Li**: Writing—original draft; writing—review and editing; validation. **Xiaoli Feng**: Supervision; resources; methodology; funding acquisition; writing—review and editing.

## CONFLICT OF INTEREST STATEMENT

The authors declare no conflicts of interest.

### PEER REVIEW

The peer review history for this article is available at https://publons.com/publon/10.1002/brb3.3636.

## Data Availability

The data that support the findings of this study are available from the corresponding author upon reasonable request.

## References

[brb33636-bib-0001] Aberg‐Wistedt, A. , Alvariza, M. , Bertilsson, L. , Malmgren, R. , & Wachtmeister, H. (1985). Alaproclate a novel antidepressant? A biochemical and clinical comparison with zimeldine. Acta Psychiatrica Scandinavica, 71, 256–268. 2580422 10.1111/j.1600-0447.1985.tb01282.x

[brb33636-bib-0002] Argyelán, M. , Szabó, Z. , Kanyó, B. , Tanács, A. , Kovács, Z. , Janka, Z. , & Pávics, L. (2005). Dopamine transporter availability in medication free and in bupropion treated depression: A 99mTc‐TRODAT‐1 SPECT study. Journal of Affective Disorders, 89, 115–123. 16213028 10.1016/j.jad.2005.08.016

[brb33636-bib-0003] Asim, M. , Wang, H. , Chen, X. , & He, J. (2024). Potentiated GABAergic neuronal activities in the basolateral amygdala alleviate stress‐induced depressive behaviors. CNS Neuroscience & Therapeutics, 30, e14422. 37715582 10.1111/cns.14422 PMC10915993

[brb33636-bib-0004] Asim, M. , Wang, H. , & Waris, A. (2023). Altered neurotransmission in stress‐induced depressive disorders: The underlying role of the amygdala in depression. Neuropeptides, 98, 102322. 36702033 10.1016/j.npep.2023.102322

[brb33636-bib-0005] Asim, M. , Wang, H. , Waris, A. , Qianqian, G. , & Chen, X. (2024). Cholecystokinin neurotransmission in the central nervous system: Insights into its role in health and disease. *Biofactors*. (Oxford, England), 22 May. 2024, 10.1002/biof.2081 PMC11627476 38777339

[brb33636-bib-0006] Brake, W. G. , Zhang, T. Y. , Diorio, J. , Meaney, M. J. , & Gratton, A. (2004). Influence of early postnatal rearing conditions on mesocorticolimbic dopamine and behavioural responses to psychostimulants and stressors in adult rats. European Journal of Neuroscience, 19, 1863–1874. 15078560 10.1111/j.1460-9568.2004.03286.x

[brb33636-bib-0007] Brenhouse, H. C. , Danese, A. , & Grassi‐Oliveira, R. (2019). Neuroimmune impacts of early‐life stress on development and psychopathology. Current Topics in Behavioral Neurosciences, 43, 423–447. 30003509 10.1007/7854_2018_53

[brb33636-bib-0008] Brown, D. W. , Anda, R. F. , Tiemeier, H. , Felitti, V. J. , Edwards, V. J. , Croft, J. B. , & Giles, W. H. (2009). Adverse childhood experiences and the risk of premature mortality. American Journal of Preventive Medicine, 37, 389–396. 19840693 10.1016/j.amepre.2009.06.021

[brb33636-bib-0009] Cai, H. , Jin, Y. , Liu, R. , Zhang, Q. , Su, Z. , Ungvari, G. S. , Tang, Y. L. , Ng, C. H. , Li, X. H. , & Xiang, Y. T. (2023). Global prevalence of depression in older adults: A systematic review and meta‐analysis of epidemiological surveys. Asian Journal of Psychiatry, 80, 103417. 36587492 10.1016/j.ajp.2022.103417

[brb33636-bib-0010] Chapman, D. P. , Whitfield, C. L. , Felitti, V. J. , Dube, S. R. , Edwards, V. J. , & Anda, R. F. (2004). Adverse childhood experiences and the risk of depressive disorders in adulthood. Journal of Affective Disorders, 82, 217–225. 15488250 10.1016/j.jad.2003.12.013

[brb33636-bib-0011] Chaudhury, D. , Walsh, J. J. , Friedman, A. K. , Juarez, B. , Ku, S. M. , Koo, J. W. , Ferguson, D. , Tsai, H. C. , Pomeranz, L. , Christoffel, D. J. , Nectow, A. R. , Ekstrand, M. , Domingos, A. , Mazei‐Robison, M. S. , Mouzon, E. , Lobo, M. K. , Neve, R. L. , Friedman, J. M. , Russo, S. J. , … Han, M. H. (2013). Rapid regulation of depression‐related behaviours by control of midbrain dopamine neurons. Nature, 493, 532–536. 23235832 10.1038/nature11713 PMC3554860

[brb33636-bib-0013] Chocyk, A. , Przyborowska, A. , Dudys, D. , Majcher, I. , Maćkowiak, M. , & Wędzony, K. (2011). The impact of maternal separation on the number of tyrosine hydroxylase‐expressing midbrain neurons during different stages of ontogenesis. Neuroscience, 182, 43–61. 21396433 10.1016/j.neuroscience.2011.03.008

[brb33636-bib-0014] Chodavadia, P. , Teo, I. , Poremski, D. , Fung, D. S. S. , & Finkelstein, E. A. (2023). Prevalence and economic burden of depression and anxiety symptoms among Singaporean adults: Results from a 2022 web panel. BMC Psychiatry [Electronic Resource], 23, 104. 36782116 10.1186/s12888-023-04581-7 PMC9925363

[brb33636-bib-0015] Cohen, H. , Kaplan, Z. , Matar, M. A. , Loewenthal, U. , Zohar, J. , & Richter‐Levin, G. (2007). Long‐lasting behavioral effects of juvenile trauma in an animal model of PTSD associated with a failure of the autonomic nervous system to recover. European Neuropsychopharmacology, 17, 464–477. 17196373 10.1016/j.euroneuro.2006.11.003

[brb33636-bib-0016] Faustman, W. O. , King, R. J. , Faull, K. F. , Moses, J. A. Jr. , Benson, K. L. , Zarcone, V. P. , & Csernansky, J. G. (1991). MMPI measures of impulsivity and depression correlate with CSF 5‐HIAA and HVA in depression but not schizophrenia. Journal of Affective Disorders, 22, 235–239. 1939932 10.1016/0165-0327(91)90069-5

[brb33636-bib-0017] Feng, X. , Wang, L. , Yang, S. , Qin, D. , Wang, J. , Li, C. , Lv, L. , Ma, Y. , & Hu, X. (2011). Maternal separation produces lasting changes in cortisol and behavior in rhesus monkeys. PNAS, 108, 14312–14317. 21844333 10.1073/pnas.1010943108 PMC3161556

[brb33636-bib-0041] Fu, R. , Jinnah, H. , McKay, J. L. , Miller, A. H. , Felger, J. C. , Farber, E. W. , Sharma, S. , Whicker, N. , Moore, R. C. , Franklin, D. , Letendre, S. L. , & Anderson, A. M. (2023). Cerebrospinal fluid levels of 5‐HIAA anddopamine in people with HIV and depression. J Neurovirol, 29, 440–448. 37289360 10.1007/s13365-023-01142-2 PMC10766341

[brb33636-bib-0018] Goff, B. , & Tottenham, N. (2015). Early‐life adversity and adolescent depression: Mechanisms involving the ventral striatum. CNS Spectrums, 20, 337–345. 25511634 10.1017/S1092852914000674 PMC5928787

[brb33636-bib-0019] Hirschfeld, R. M. (2000). History and evolution of the monoamine hypothesis of depression. Journal of Clinical Psychiatry, 61(Suppl 6), 4–6. 10775017

[brb33636-bib-0020] Hunt, T. K. A. , Slack, K. S. , & Berger, L. M. (2017). Adverse childhood experiences and behavioral problems in middle childhood. Child Abuse & Neglect, 67, 391–402. 27884508 10.1016/j.chiabu.2016.11.005 PMC5436949

[brb33636-bib-0021] Jahng, J. W. , Ryu, V. , Yoo, S. B. , Noh, S. J. , Kim, J. Y. , & Lee, J. H. (2010). Mesolimbic dopaminergic activity responding to acute stress is blunted in adolescent rats that experienced neonatal maternal separation. Neuroscience, 171, 144–152. 20828601 10.1016/j.neuroscience.2010.08.063

[brb33636-bib-0022] Jiang, Y. , Zou, D. , Li, Y. , Gu, S. , Dong, J. , Ma, X. , Xu, S. , Wang, F. , & Huang, J. H. (2022). Monoamine neurotransmitters control basic emotions and affect major depressive disorders. Pharmaceuticals (Basel), 15(10), 1203. 36297314 10.3390/ph15101203 PMC9611768

[brb33636-bib-0023] Jori, A. , Dolfini, E. , Casati, C. , & Argenta, G. (1975). Effect of ECT and imipramine treatment on the concentration of 5‐hydroxyindoleacetic acid (5HIAA) and homovanillic acid (HVA) in the cerebrospinal fluid of depressed patients. Psychopharmacologia, 44, 87–90. 1105628 10.1007/BF00421189

[brb33636-bib-0024] López‐Muñoz, F. , & Alamo, C. (2009). Monoaminergic neurotransmission: The history of the discovery of antidepressants from 1950s until today. Current Pharmaceutical Design, 15, 1563–1586. 19442174 10.2174/138161209788168001

[brb33636-bib-0025] Lopez, A. D. , & Murray, C. C. (1998). The global burden of disease, 1990–2020. Nature Medicine, 4, 1241–1243. 10.1038/3218 9809543

[brb33636-bib-0026] Malhi, G. S. , & Mann, J. J. (2018). Depression. Lancet, 392, 2299–2312. 30396512 10.1016/S0140-6736(18)31948-2

[brb33636-bib-0027] Marwaha, S. , Palmer, E. , Suppes, T. , Cons, E. , Young, A. H. , & Upthegrove, R. (2023). Novel and emerging treatments for major depression. Lancet, 401, 141–153. 36535295 10.1016/S0140-6736(22)02080-3

[brb33636-bib-0028] Mcewen, B. S. , & Morrison, J. H. (2013). The brain on stress: Vulnerability and plasticity of the prefrontal cortex over the life course. Neuron, 79, 16–29. 23849196 10.1016/j.neuron.2013.06.028 PMC3753223

[brb33636-bib-0029] Meador‐Woodruff, J. H. , Damask, S. P. , & Watson, S. J. Jr. (1994). Differential expression of autoreceptors in the ascending dopamine systems of the human brain. PNAS, 91, 8297–8301. 7914704 10.1073/pnas.91.17.8297 PMC44593

[brb33636-bib-0030] Nemeroff, C. C. (2004). Early‐life adversity, CRF dysregulation, and vulnerability to mood and anxiety disorders. Psychopharmacology Bulletin, 38, 14–20. 15278013

[brb33636-bib-0031] Ogawa, S. , & Kunugi, H. (2019). Evidence for reduced homovanillic acid (HVA) in the cerebrospinal fluid of patients with depression. Journal of Affective Disorders, 255, S0165–S0327. 10.1016/j.jad.2019.04.028 31006502

[brb33636-bib-0032] Ogawa, S. , Tsuchimine, S. , & Kunugi, H. (2018). Cerebrospinal fluid monoamine metabolite concentrations in depressive disorder: A meta‐analysis of historic evidence. Journal of Psychiatric Research, 105, 137–146. 30219563 10.1016/j.jpsychires.2018.08.028

[brb33636-bib-0033] Oswald, L. M. , Wand, G. S. , Kuwabara, H. , Wong, D. F. , Zhu, S. , & Brasic, J. R. (2014). History of childhood adversity is positively associated with ventral striatal dopamine responses to amphetamine. Psychopharmacology, 231, 2417–2433. 24448898 10.1007/s00213-013-3407-z PMC4040334

[brb33636-bib-0034] Post, R. M. , Rubinow, D. R. , Uhde, T. W. , Ballenger, J. C. , & Linnoila, M. (1986). Dopaminergic effects of carbamazepine. Relationship to clinical response in affective illness. Archives of General Psychiatry, 43, 392–396. 3954558 10.1001/archpsyc.1986.01800040102014

[brb33636-bib-0035] Scheinin, M. , Chang, W. H. , Kirk, K. L. , & Linnoila, M. (1983). Simultaneous determination of 3‐methoxy‐4‐hydroxyphenylglycol, 5‐hydroxyindoleacetic acid, and homovanillic acid in cerebrospinal fluid with high‐performance liquid chromatography using electrochemical detection. Analytical Biochemistry, 131, 246–253. 6193730 10.1016/0003-2697(83)90162-8

[brb33636-bib-0036] Strittmatter, W. J. (2013). Bathing the brain. Journal of Clinical Investigation, 123, 1013–1015. 23434595 10.1172/JCI68241 PMC3582153

[brb33636-bib-0037] Van Wingen, G. A. , Tendolkar, I. , Urner, M. , Van Marle, H. J. , Denys, D. , Verkes, R. J. , & Fernández, G. (2014). Short‐term antidepressant administration reduces default mode and task‐positive network connectivity in healthy individuals during rest. Neuroimage, 88, 47–53. 24269575 10.1016/j.neuroimage.2013.11.022

[brb33636-bib-0038] Venzala, E. , García‐García, A. L. , Elizalde, N. , & Tordera, R. M. (2013). Social vs. environmental stress models of depression from a behavioural and neurochemical approach. European Neuropsychopharmacology, 23, 697–708. 22743048 10.1016/j.euroneuro.2012.05.010

[brb33636-bib-0039] Zhang, Z. Y. , Mao, Y. , Feng, X. L. , Zheng, N. , Lü, L. B. , Ma, Y. Y. , Qin, D. D. , & Hu, X. T. (2016). Early adversity contributes to chronic stress induced depression‐like behavior in adolescent male rhesus monkeys. Behavioural Brain Research, 306, 154–159. 27025444 10.1016/j.bbr.2016.03.040

